# Supplementary material for: Effectiveness of a Nutrition Education Programme on Nutritional Knowledge in Young Football Players: A Pilot Study
Source: Nutrients. 2025 Jul 23;17(15):2404. doi: 10.3390/nu17152404 (PMC12348160; doi:10.3390/nu17152404)
Supplement: Supplementary file 1 [file nutrients-17-02404-s001.zip › nutrients-3758954-supplementary.pdf]

CÓDIGO DO CLUBE
XXYY

CÓDIGO DO PARTICIPANTE
MESANOINICIAIS

PGENSK: QUESTIONÁRIO SOBRE CONHECIMENTOS SOBRE ALIMENTAÇÃO E NUTRIÇÃO[2]

O questionário que se segue pretende saber o que sabes sobre alimentos, nutrientes e alimentação para o desporto.
Se não souberes a resposta podes selecionar a opção "Não sei".

Secção 1 – Nutrição

1 - O teor de hidratos de carbono dos seguintes alimentos é:

	ALTO	BAIXO/NENHUM	NÃO SEI
Fiambre			
Tomate			
Maçã			
Queijo flamengo			

2 – O teor em proteína dos seguintes alimentos é:

	ALTO	BAIXO/NENHUM	NÃO SEI
Pera			
Arroz			
Bacalhau			
Queijo			
Chocolate			

3 – O teor em gordura dos seguintes alimentos é:

	ALTO	BAIXO/NENHUM	NÃO SEI
Compota			
Grão de bico			
Manteiga			
Maionese			
Massa cozida			

4 – O teor de sal dos seguintes alimentos é:

	ALTO	BAIXO/NENHUM	NÃO SEI
Pão			
Atum em lata			

DO NOT COPY WITHOUT PERMISSION

Ervilhas em lata			

Ervilhas congeladas			
Courgete			

5 – O teor de cálcio dos seguintes alimentos é:

	ALTO	BAIXO/NENHUM	NÃO SEI
Ervilhas			
Pão			
Peito de peru			
Azeite			
Brócolos			

6 – O teor de ferro dos seguintes alimentos é

	ALTO	BAIXO/NENHUM	NÃO SEI
Peixe			
Fígado (iscas)			
Feijão			
Mel			

7 – Considerando o rótulo apresentado abaixo,

	Por 100g
Açúcar	8,8
Gordura total	0,9
Dos quais ácidos gordos saturados	0,08
Sal	0,215

a) Como classificas a quantidade de açúcar presente neste produto? Assinala com um X.

ALTO	BAIXO	MÉDIO	NÃO SEI

a) Como descreves a quantidade de gordura presente no alimento? Assinala com um X.

ALTO	BAIXO	MÉDIO	NÃO SEI

DO NOT COPY WITHOUT PERMISSION

b) Como descreves o teor de ácidos gordos presente no alimento? Assinala com um X.

ALTO	BAIXO	MÉDIO	NÃO SEI

a) Como descreves o teor de sal presente no alimento? Assinala com um X.

ALTO	BAIXO	MÉDIO	NÃO SEI

8 – Como está apresentada a lista de ingredientes no rótulo de um alimento?

ORDEM CRESCENTE	ORDEM DECRESCENTE	NÃO SEI

9 – É obrigatório que a lista de ingredientes tenha a % de cada ingrediente?

SIM	NÃO	NÃO SEI

Secção 2 – Nutrição no desporto

10 - Classifica cada uma das seguintes afirmações como Verdadeira ou Falsa:

	VERDADEIRA	FALSA	NÃO SEI
Ingerir hidratos de carbono é bom para o atleta			
Os atletas devem reduzir a ingestão de gordura ao mínimo possível			
Ingerir mais proteína aumenta a massa muscular			
Os atletas podem comer o que quiserem porque gastam muito nos treinos			
Precisamos de açúcar para ter energia			
O atleta deve ingerir o dobro da proteína que um indivíduo sedentário			
Os atletas devem beber apenas quando têm sede			

DO NOT COPY WITHOUT PERMISSION

É necessário reduzir a ingestão de pão, arroz e massa numa fase de treinos			
Depois de treinar posso comer o que quiser			

Assinala a resposta certa

11 – Quanto tempo deve comer antes de uma sessão de treino?

a) 5 a 10 minutos antes
b) 30 minutos
c) Pelo menos 1h antes
d) Não sei

12 – Qual a opção mais correta para uma refeição antes do treino?

a) Leite com cereais tipo corn flakes
b) Um bolo e um leite com chocolate
c) Uma sandes e uma peça de fruta
d) Não sei

13 – Qual a principal fonte de energia durante o treino?
a) Gordura
b) Proteínas
c) Hidratos de carbono
d) Não sei

14 – Se tiveres fome e falta de energia 5-10 minutos antes de um treino podes comer:
a) Um chocolate
b) Um ovo cozido
c) Uma barra de cereais
d) Não sei

Muito obrigada pela colaboração!

DO NOT COPY WITHOUT PERMISSION
